# Sensibilité aux antibiotiques des souches de*staphylococcus aureus* communautaires dans la région de Nouakchott (Mauritanie)

**DOI:** 10.11604/pamj.2016.24.276.9865

**Published:** 2016-07-27

**Authors:** Mohamed Lemine Ould Salem, Sidi Mohamed Ghaber, Sidi El Wafi Ould Baba, Mohamed Mahmoud Ould Maouloud

**Affiliations:** 1 Service des Laboratoires, Centre Hospitalier National de Nouakchott, Mauritanie; 2 Faculté de Médecine de Nouakchott, Mauritanie; 3 Service de Médecine Interne, Centre Hospitalier National de Nouakchott, Mauritanie

**Keywords:** Staphylococcus aureus, SARM, résistance aux antibiotiques, Nouakchott, Mauritanie, Staphylococcus aureus, MRSA, antibiotic resistance, Nouakchott, Mauritania

## Abstract

**Introduction:**

*Staphylocoque aureus* reste un pathogène majeur de l'homme causant des infections très diverses, cutanées, urinaires, pulmonaires ainsi que des septicémies. L'objectif de ce travail est d'évaluer la sensibilité des souches communautaires de *Staphylococcus aureus* isolées dans différents produits pathologiques vis-à-vis des principaux antibiotiques utilisés, dans la région de Nouakchott (Mauritanie).

**Méthodes:**

Il s'agit d'une étude rétrospective réalisée sur 281 souches de *Staphylococus aureus* isolées entre Janvier 2014 et Août 2015 au laboratoire du Centre Hospitalier National et aux deux laboratoires privés de la ville de Nouakchott, dans différents produits pathologiques des patients non hospitalisés. La sensibilité aux antibiotiques a été déterminée par la méthode de diffusion de disques en milieu gélosé de Mueller Hinton selon les recommandations du CA-SFM.

**Résultats:**

Le taux de résistance à la pénicilline G était élevé (96 à 100%). Le taux de SARM communautaires se situe entre 25 et 26% dans les suppurations, de 34,3% dans les ECBU et de 28% dans les spermocultures. La résistance aux Macrolides-Lincosamyne-Streptogramines (MLS), donnant le phénotype MLSb inductible, était retrouvée dans 6% des souches urinaires et 27% des souches isolées à partir des suppurations. L'activité des aminosides est variable, l'amikacine était active sur toutes les souches. L'activité du cotrimoxazol est faible (77% de résistance) et aucune résistance à la vancomycine n'a été notée.

**Conclusion:**

L'activité de la Pénicilline G sur les souches de *Staphylococcus aureus* isolées dans la région de Nouakchott est quasi nulle et le taux de SARM communautaire est important atteignant jusqu'à 34%. Ceci pourrait être expliqué par l'usage anarchique de ces molécules dans notre pays.

## Abstract (en)

**Introduction:**

Staphilococcus aureus is a leading pathogen for humans causing a variety of infections such as skin, urinary tract and lung infections as well as sepsis. This study aims to evaluate the susceptibility of community-acquired strains of Staphylococcus aureus, isolated from various pathological products, compared with major antibiotics used in Nouakchott Region (Mauritania).

**Methods:**

We conducted a retrospective study of 281 strains of Staphylococcus aureus strains isolated from various pathological products from non-hospitalized patients in the National referral hospital laboratory and in two private laboratories in the city of Nouakchott between January 2014 and August 2015. Antibiotic sensitivity was determined by disk diffusion method using agar containing Mueller-Hinton medium according to CA-SFM's recommendations.

**Results:**

The resistance rate to penicillin G was high (96-100%). Community-acquired MRSA rate was between 25 and 26% in suppurations, 34.3% in urine cultures and 28% in sperm cultures. Macrolide -Lincosamyne-streptogramins (MLS) resistance, giving rise to the phenotype MLSb inducible, was found in 6% of urinary strains and 27% of strains isolated from suppurations. The activity of aminoglycosides was variable, amikacin was active against all strains. Cotrimoxazole activity was low (77% had resistance) and no vancomycin resistance was reported.

**Conclusion:**

The activity of penicillin G against Staphylococcus aureusstrains isolated in Nouakchott region is almost zero and community-acquired MRSA rate is high, accounting for 34%. This could be explained by uncontrolled use of these molecules in our country.

## Introduction

*Staphylococus aureus (S. aureus)*, décrit pour la première fois par Ogston il y'a plus de cent ans, reste un pathogène majeur de l'homme, causant des infections de la peau et des tissus mous, des septicémies, des pneumonies, des endocardites et des abcès profonds [1, 2]. C'est également le germe le plus isolé dans les infections des plaies par des corps étrangers [1] et est responsable également de toxi-infections alimentaires [2]. Cette bactérie est devenue de plus en plus résistante aux antibiotiques; en effet, la résistance à la Pénicilline a rapidement atteint les 90% des souches. C'est au début des années 1960 que les premières souches de *S. aureus* résistantes à la méticilline (SARM) sont apparues après introduction de la méticilline, première bêta-lactamine résistante aux pénicillinases [1]. La résistance à la méticilline est liée à l'acquisition d'une autre protéine liant la pénicilline, la PLP2a ou PLP2', présentant peu d'affinité pour les béta-lactamines. La production de PLP2a est codée par le gène chromosomique *mec A* dont l'origine reste encore inconnue [3]. La fréquence des SARM est variable dans le monde, faible en France et importante aux USA atteignant jusqu'à 72% dans les infections cutanées [4]. L'objectif de ce travail est d'évaluer la sensibilité des souches communautaires de *S. aureus* isolées à partir de différents produits pathologiques vis-à-vis des principaux antibiotiques utilisés dans le traitement des infections dues à ce germe, dans la région de Nouakchott en Mauritanie.

## Méthodes

**Souches étudiées:** Il s'agit d'une étude rétrospective qui a été réalisée sur l'ensemble des souches de*Staphylococus aureus* isolés entre Janvier 2014 et Août 2015 au laboratoire du Centre Hospitalier National et au deux laboratoires privés de la ville de Nouakchott, dans différents produits pathologiques (Tableau 1). Il s'agit au total de 281 souches dont l'identification a été faite selon les critères classiques : morphologie des colonies, coloration de Gram, recherche de la catalase et de la coagulase et fermentation du mannitol.

**Tableau 1:** Nombre de souches par type de prélèvement

Nature du prélèvement	Nombre de souches
Suppuration cutanée	175
Pus d’otite moyenne chronique	35
Examen cytobactériologique des urines (ECBU)	28
Spermoculture	25
Liquide synovial	12
Liquide pleural	2
Ecouvillonnage nasal	2
Prélèvement urétral	2
**Total**	**281**

**Sensibilité aux antibiotiques:** La sensibilité aux antibiotiques a été déterminée par la méthode de diffusion de disques en milieu gélosé de Muller/Hinton, selon les recommandations du CA-SFM (comité de l'antibiogramme de la société française de Microbiologie) [5]. Les antibiotiques étudiés sont : pénicilline G (P-6µg), oxacilline (OXA-5mg), fosfomycine (FOS-5mg), érythromycine (E-15UI), chloramphénicol (C-30mg), lincomycine (L-15mg), gentamicine (GM-15mg), spiramycine (SP-100mg), ofloxacine (OFX-5mg), ciprofloxacine (CIP-5mg), amikacine (AN-30UI), vancomycine (VA-30mg) et cefoxitine (FOX-30mg), La recherche de la résistance à la méticilline a été effectuée par la méthode de diffusion, d'une part du disque de céfoxitine 30 µg où un diamètre d'inhibition autour du disque de moins de 27 mm et la présence de colonies dans la zone d'inhibition témoignent de la présence d'un SARM, et d'autre part, par le disque d'oxacilline 5 mg où la présence d'une souche ayant un diamètre < 20 mm est considérée comme SARM.

## Résultats

Cette étude concernait les souches de *S aureus* isolées à partir de 281 produits pathologiques, dont les suppurations cutanées étaient majoritaires représentant ainsi 62.6% (157/281) des produits pathologiques, suivies des suppurations d’otites moyennes chroniques : 12.4% (35/281), des ECBU : 10% (28/281), des spermocultures : 8.9% (25/281) et des liquides articulaires : 4.2% (12/281). La bactérie a été également isolée dans d’autres produits pathologiques tels que le liquide pleural, l’écouvillonnage nasal et le prélèvement urétral. La répartition des souches de S aureus, selon les différents produits pathologiques est représentée dans le Tableau 1.

Concernant les résultats de la résistance aux antibiotiques des souches de *S aureus*, ne sont rapportés que ceux concernant les souches isolées des produits pathologiques où le nombre était significatif (les suppurations cutanées, les suppurations d’otites moyennes chroniques, les ECBU, les spermocultures, et les liquides articulaires). L’antibiotique présentant la plus faible activité sur les souches isolées à partir des différents produits pathologiques était la pénicilline (96 à 100% de résistance), suivi du cotrimoxazol (66 à 75% de résistance). La vancomycine et l’amikacine étaient, en revanche, actives sur l’ensemble des souches isolées. La fréquence de résistance aux antibiotiques étudiés des différentes souches isolées selon l'origine de l'infection est représentée sans le Tableau 2.

**Tableau 2:** Fréquence de résistance aux antibiotiques par produit pathologique

Antibiotique	P	0XA	FOS	E	C	L	GM	SP	OFX	CIP	AN	SXT	VA
Suppurations cutanées	97,2	26,3	8,57	31,4	48	2,85	6,85	7,8	0	0	0		0
otites	97,1	25,7	8,5	31,4	51,4	2,9	5,8	8,7	5,8	5,8	0		0
Liquide synovial	100	25	8,3	33,3	41,6	8,3	8,3	0	0	0	0	66,6	0
ECBU	96,4	34,3	14,2	10,7	50	3,5	7,1	7,1	10,7	10,7	0	75	0
Spermocultures	96,0	28	7	40	64	4	8	8	4	4	0	72	0

## Discussion

Le taux de souches sauvages dans notre série était faible environ 3%. Elazhari et al. [1] décrivent au Maroc, un taux comparable d'environ 4%. La résistance à la pénicilline G dans notre série est importante dépassant celle décrite par plusieurs auteurs [1, 6, 7]. Le phénotype Pénicilline G isolé était présent en moyenne de 37% dans les suppurations. Le taux de SARM communautaires qui était entre 25% et 26% dans les suppurations, de 34,3% dans les ECBU et de 28% dans les spermocultures est plus élevé que celui décrit par El azhari et al. [1] qui rapportent un taux seulement de 7,1% à Casablanca et de celui décrit par El hamzaoui [8] et al. (7 souches sur 61, soit 11,4%) chez les patients externes. Ce taux important des SARM communautaires pourrait être expliqué par l'usage anarchique de l'oxacilline dans notre pays. La résistance aux Macrolides-Lincosamyne-Streptogramines (MLS) donnant le phénotype MLSb inductible, était retrouvée dans 6% des souches urinaires et 27% des souches isolées à partir des suppurations. Ces taux importants de résistance pourraient s'expliquer eux aussi par l'usage inapproprié de l'érythromycine.

Le phénotype MLSb constitutif représentait 2% au sein des isolats urinaires et environ 3% dans les suppurations. Les fluoroquinolones gardent une bonne activité sur les souches de pus. En revanche, un taux non négligeable de résistance à été constaté avec les souches isolées des ECBU dont l'usage de ces molécules en antibiothérapie probabiliste des infections urinaires pourrait en être la cause. L'activité des aminosides est variable, l'amikacine était active sur toutes les souches (cette molécule n'est pas commercialisée en Mauritanie) alors que la résistance à la gentamicine est de d'ordre de 10% et souvent associée au phénotype SARM. Le cotrimoxazol est quasi inactif (77% de résistance). Aucune résistance à la vancomycine n'a été retrouvée.

## Conclusion

L'activité de la Pénicilline G sur les souches de *S. aureus* isolées dans la région de Nouakchott est quasi nulle et le taux de SARM communautaire est important atteignant jusqu'à 34%. Ceci pourrait être expliqué par l'usage anarchique de ces molécules ce phénomène doit inciter les autorités sanitaires du pays à envisager une stratégie efficace concernant l'approvisionnement et les règles de prescription des ces molécules. Notons que la spiramycine et la lincomycine gardent une bonne activité sur les souches de s aureus et pourraient constituer par conséquent une alternative pour le traitement de ces infections.

### Etat des connaissances actuelles sur le sujet

- *Staphylococcus aureus* reste le germe le plus fréquent au cours des infections cutanées communautaires;
- L'émergence des staphylocoques résistants à la méticilline (SARM) en milieu communautaire est actuellement devenue une donnée bien connue dans le monde.

### Contribution de notre étude à la connaissance

- Il s'agit de la première étude mauritanienne décrivant la fréquence des *Staphylococcus aureus* dans différents produits pathologiques ainsi que leur sensibilité aux antibiotiques en milieu communautaire.
